# Understanding Family Caregiver Experience and Satisfaction with Nursing Home Care: A Conceptual Model

**DOI:** 10.1002/alz.083710

**Published:** 2025-01-09

**Authors:** Catherine V Piersol, Jenny Martinez, Felicia M Chew, Natalie E Leland

**Affiliations:** ^1^ Thomas Jefferson University, Philadelphia, PA USA; ^2^ University of Pittsburgh, Pittsburgh, PA USA

## Abstract

**Background:**

Family caregivers are challenged to manage the progressive cognitive decline and behavioral symptoms of Alzheimer’s disease and related dementias, which often trigger nursing home placement. Research has addressed the effect of nursing home placement on family caregivers; however, their perceptions about care are less understood. We sought to examine the experiences of family caregivers with the objective of developing a conceptual model to explain the complex nature of family caregiver experience.

**Methods:**

We will describe our qualitative study that utilized the grounded theory method. Purposive sampling was used to recruit family caregivers from a sample of 23 nursing homes in the United States enrolled in a larger pragmatic trial. Caregivers participated in one‐on‐one, in‐depth interviews with trained research assistants. An iterative, three‐step data coding process was employed to analyze the interview transcripts.

**Results:**

Study participants (N = 30) were primarily female (90%), White (86.6%), and ranged from age 29 to 84. This presentation will characterize seven emerging themes that interrelate to create a conceptual model. One theme, “Family caregiver satisfaction with care” emerged as the core concept, with the other six themes serving as contributing intrinsic and extrinsic factors that interrelate and impact family caregiver perception of satisfaction. The contributing factors are (1) family caregiver interactions with nursing home staff, (2) staff management of resident behavioral symptoms; (3) nursing home context; (4) family caregiver knowledge of dementias; (5) family caregiver strain; and (6) the resident experience. Family caregivers experience each contributor simultaneously and in differing patterns, depending on the care situation. We will display our conceptual model and discuss how the interactions between contributing factors affect family caregiver satisfaction across a dynamic continuum from low to high satisfaction.

**Conclusion:**

Our conceptual model offers a mechanism for understanding the factors important to family caregivers of nursing home residents with dementia, prompting their action and reaction to care situations and staff interactions. The model reflects the importance caregivers place on staff knowledge about dementia and their use of appropriate care approaches. Understanding how satisfaction with care is perceived by family caregivers may encourage collaboration between families and staff.

